# Early Steroid Withdrawal in Kidney Transplant Recipients: COMMENTARY

**DOI:** 10.34067/KID.0000000571

**Published:** 2025-02-27

**Authors:** Dixon B. Kaufman

**Affiliations:** Department of Surgery, School of Medicine and Public Health, University of Wisconsin-Madison, Madison, Wisconsin

**Keywords:** acute rejection, clinical immunology, immunosuppression, kidney transplantation, renal transplantation, transplant outcomes

Corticosteroids are the most versatile, frequently used, longest-standing, inexpensive, and controversial medications prescribed for transplant immunosuppression. While these drugs have undeniable benefits as induction agents, maintenance agents, and antirejection therapies, their adverse side effect profile have prompted ongoing debates regarding their long-term use; optimal dosages; and whether, in whom, and when they can be eliminated from maintenance therapy treatment protocols.

Traditionally, corticosteroids are administered at high doses during the initial post-transplant period (little controversy), followed by a taper to lower maintenance doses over a period as short as 3 days (rapid elimination), a week or so (rapid taper and elimination), or continued indefinitely and slowly tapered over 1–3 months to a final dose in the range of 5–10 mg per day. Despite their effectiveness, corticosteroids are associated with a wide range of side effects, most of which being time dependent and irreversible. In the very short term, during the induction phase with high dosing, early post-transplant hyperglycemia requiring temporary exogenous insulin therapy not infrequently occurs. Hypertension and fluid weight gain are also temporarily exacerbated. These resolve reasonably quickly during the index hospital stay (sometimes prolonging hospitalization) and early post-transplant out-patient course because the dosage rapidly decreases or the medications stopped.

Where the crux of the controversy centers is the long-term and continuous use of corticosteroids throughout the patient's lifespan with a transplanted organ. This is where the risks, most irreversible, caused by chronic corticosteroid use becomes more concerning to patients: weight gain, hypertension, diabetes, osteoporosis, cardiovascular disease, sleeplessness, anxiety, and an increased susceptibility to infections. The chronic use of corticosteroids can also lead to Cushing syndrome, characterized by symptoms like moon face, fat redistribution, and skin changes and growth retardation in children. In years past when less effective combinations of maintenance immunosuppressive agents were available, corticosteroids were a critical component (*e.g*., azathioprine and prednisone; azathioprine, cyclosporine, and prednisone), and the side effects were the necessary trade-offs for avoiding rejection and graft loss to live a life free from chronic dialysis (or death). However, as stronger and more effective immunosuppressive therapeutics were developed (including biologics) and anti-infectious prophylaxis and therapeutics evolved, new combination maintenance immunosuppression alternatives were developed as used today.

The question naturally arose whether continuous corticosteroid use was still needed and worth the tradeoffs of side effects and diminished quality of life. This led to a very active period of single-center and, eventually, multicenter, randomized clinical trials and registry reports, examining the outcomes of corticosteroid-sparing and corticosteroid-free regimens, as referenced in the PRO and CON sections.^1,2^ One of the difficulties in addressing the merits of the debating positions relates to the context of the clinical scenario being evaluated. This can introduce complexity in trying to settle the debate when it is unrecognized that the different sides are examining different aspects of the multitude of variations pertaining to steroid-sparing. Agreeing where to focus is an important beginning.

## Corticosteroid-Sparing and Corticosteroid-Free Regimens: The Debate

The move toward eliminating corticosteroids in kidney transplantation had been tested before the availability of tacrolimus, mycophenolate mofetil, and mammalian target of rapamycin inhibitors. It did not go well. One of the most concerning reports of the failure of late corticosteroid withdrawal still exerts a sobering effect on the judgment of steroid elimination protocols.^3^ In fact, late withdrawal is probably not controversial and is still considered by most practitioners to be considered inadvisable. Therefore, the emphasis has focused on two corticosteroid elimination approaches. It is between those choices versus continuous corticosteroid use where the debate is most relevant.

The controversy lies in balancing the benefits of reducing corticosteroid exposure with the risks of underimmunosuppression. When considering risks in this context, it is important to differentiate the risk of a rejection episode (relevant) from that of immediate graft loss (rare). While some trials demonstrate that these regimens can reduce side effects without significantly increasing rejection rates, others report elevated incidences of acute rejection, especially in high-risk patients. The higher risk recipients may include retransplant recipients, highly sensitized recipients, pancreas-kidney transplant recipients, and those with an underlying cause of nephropathy from autoimmune etiologies.

Another aspect of the debate revolves around how to apply corticosteroid elimination, if at all. Minimizing the risk of early rejection in recipients receiving a corticosteroid rapid elimination immunosuppression approach is commonly prevented by pairing it with a strong antilymphocyte depletion induction therapy approach using antilymphocyte globulin or alemtuzumab. However, there are several recipient populations in which lymphocyte depletion is a relative contraindication due to risk of overimmunosuppression. These groups include those at advanced age with or without significant comorbidities or retransplant recipients having been exposed to maintenance immunosuppression for many years. These complexities of immunosuppression management challenge a one-size-fits-all approach and is not at the center of the debate.

## Evidence-Based Clinical Trials

Numerous clinical trials have examined the outcomes of corticosteroid-sparing and corticosteroid-free protocols that support both positions of continuation or discontinuation, as nicely referenced by our contributors. Given the mixed evidence, professional guidelines for corticosteroid use in kidney transplantation remain somewhat varied. Early on (year 2000), the Kidney Disease Improving Global Outcomes guidelines recommended a cautious approach to corticosteroid withdrawal, emphasizing that such strategies should only be considered in low-risk patients with close monitoring.^4^ Later on, there were meta-analyses, registry studies, multicenter studies, and single-center experiences that described successful approaches in many recipient subpopulations.^5^ There are now studies pushing beyond steroid withdrawal that also include calcineurin inhibitor avoidance.^6^ However, in many transplant centers today, a middle-ground approach is commonly used: Corticosteroids are administered during the early post-transplant period, followed either by gradual tapering over 1–3 months and continuation at a low dose (*e.g*., 5 mg daily) or early discontinuation between 3 and 7 days post-transplant in selected patients. These approaches attempt to leverage the benefits of corticosteroids in preventing early rejection while minimizing long-term exposure and its associated complications.

As the controversy around corticosteroid use continues, the future may lie in more personalized approaches to immunosuppressive therapy. Biomarkers and genetic profiling are established tools that help identify which patients are at higher risk for rejection and which are more likely to tolerate corticosteroid minimization. For example, the development of noninvasive tests that monitor immune activity, such as donor-derived cellfree DNA and gene expression profiling, could allow for more precise tailoring of immunosuppressive regimens. These advancements could lead to a new era where corticosteroid use is determined not by a one-size-fits-all approach, but by individual patient profiles. This would potentially allow for more effective management of immunosuppression, reducing side effects while minimizing the risk of rejection.

## Conclusion

Kidney transplantation is a life-saving and life-enhancing procedure for patients suffering from ESKD. It involves a complex array of medical management approaches, particularly in preventing organ rejection that presumes achieving years of functional outcomes for each patient. The controversy surrounding corticosteroid use in kidney transplantation reflects the challenge of balancing efficacy and safety in immunosuppressive therapy that will meet our and our patients' expectations. The body of evidence surrounding corticosteroid use in kidney transplantation is vast and nuanced. While corticosteroid-free and corticosteroid-sparing regimens offer the promise of fewer side effects and improved quality of life, they may also carry a heightened risk of acute rejection, depending on the multimodal immunosuppression approach and in which population of recipients it is being used. The decision to pursue such regimens must be individualized, taking into account the immunological risk, available monitoring tools, and the overall treatment goals. This involves considerations of critical factors of, and beyond, the immunosuppression that affect rejection and graft loss risk. Such additional considerations include health care literacy, social support structures, ability to stay adherent, distance from the transplant center, the centers' abilities to closely follow the recipient throughout the lifespan of their allograft, *etc.* As research continues to evolve and new agents are developed, it is likely that more personalized and precise approaches to corticosteroid management will emerge, potentially resolving some of the long-standing controversies in this critical area of kidney transplantation. While corticosteroids remain a cornerstone of post-transplant management, the desire to reduce their long-term side effects has spurred the development of alternative regimens. The ongoing debate is unlikely to be resolved soon because it involves complex considerations, including individual patient risk, emerging therapies, and evolving evidence. Ultimately, the decision to use, taper, or withdraw corticosteroids must be personalized, weighing the potential benefits against the risks that are understood for and by each patient.
